# Automatic initial and final segmentation in cleft palate speech of Mandarin speakers

**DOI:** 10.1371/journal.pone.0184267

**Published:** 2017-09-19

**Authors:** Ling He, Yin Liu, Heng Yin, Junpeng Zhang, Jing Zhang, Jiang Zhang

**Affiliations:** 1 School of Electrical Engineering and Information, Sichuan University, Chengdu, China; 2 Department of Cleft Lip and Palate, Hospital of Stomatology, Sichuan University, Chengdu, China; University of Akron, UNITED STATES

## Abstract

The speech unit segmentation is an important pre-processing step in the analysis of cleft palate speech. In Mandarin, one syllable is composed of two parts: initial and final. In cleft palate speech, the resonance disorders occur at the finals and the voiced initials, while the articulation disorders occur at the unvoiced initials. Thus, the initials and finals are the minimum speech units, which could reflect the characteristics of cleft palate speech disorders. In this work, an automatic initial/final segmentation method is proposed. It is an important preprocessing step in cleft palate speech signal processing. The tested cleft palate speech utterances are collected from the Cleft Palate Speech Treatment Center in the Hospital of Stomatology, Sichuan University, which has the largest cleft palate patients in China. The cleft palate speech data includes 824 speech segments, and the control samples contain 228 speech segments. The syllables are extracted from the speech utterances firstly. The proposed syllable extraction method avoids the training stage, and achieves a good performance for both voiced and unvoiced speech. Then, the syllables are classified into with “quasi-unvoiced” or with “quasi-voiced” initials. Respective initial/final segmentation methods are proposed to these two types of syllables. Moreover, a two-step segmentation method is proposed. The rough locations of syllable and initial/final boundaries are refined in the second segmentation step, in order to improve the robustness of segmentation accuracy. The experiments show that the initial/final segmentation accuracies for syllables with quasi-unvoiced initials are higher than quasi-voiced initials. For the cleft palate speech, the mean time error is 4.4ms for syllables with quasi-unvoiced initials, and 25.7ms for syllables with quasi-voiced initials, and the correct segmentation accuracy P_30_ for all the syllables is 91.69%. For the control samples, P_30_ for all the syllables is 91.24%.

## Introduction

Cleft Palate (CP) is a common congenital malformation caused by craniofacial alternation. It brings to serious dysfunctions especially in the speech intelligibility. The assessment of CP speech disorder is essential during the whole treatment of cleft palate. The speech-language pathologist is the key and unarguable member in a cleft palate care team. Currently, the perceptual assessment provided by the experienced speech-language pathologists, is the gold standard for assessment of CP speech disorders. However, it strongly depends on their personal experience. The automatic evaluation of CP speech could provide an objective aided diagnosis to both doctors and CP patients.

The clinical symptoms of CP speech are similar in almost all the languages, including resonance disorders and articulation disorders. Hypernasality is the most common resonance disorder in CP patients. It occurs at the voiced phonemes. The articulation disorders occur at the consonants. The types of articulation disorders are various, including consonant omission, consonant substitution and consonant distortion. In the processing of CP speech, the utterances are usually segmented into speech units firstly, which could reflect resonance or articulation disorders of CP speech. Thus, the complementation of automatic speech unit segmentation is an important preprocessing step in analyzing and processing CP speech.

Mandarin is the official language in China. Mandarin sounds have some significant differences from other languages. One Chinese character is a syllable, which is composed of two parts: initial and final. In cleft palate speech, the resonance disorders occur at finals and voiced initials, while the articulation disorders occur at initials. In Mandarin, initials and finals are the minimum speech units, which could reflect the characteristics of speech disorders in CP speech. It makes the Initial/Final (I/F) segmentation an important preprocessing step in CP speech processing system.

In this work, the I/F segmentation is implemented in two steps: syllable segmentation and I/F segmentation. The speech samples are segmented into syllables firstly, then for each syllable, the I/F segmentation methods are proposed. In Mandarin, syllable is the most widely used segmentation unit [1], especially in the application of automatic speech recognition and text-to-speech synthesis systems. Compared with syllable segmentation, the I/F segmentation are less studied in Mandarin.

Syllable segmentation: The most popular Chinese syllable segmentation techniques are modeling approaches based on Hidden Markov Model (HMM) [2–5], Neural Network (NN) [6–10], or Gaussian Mixture Model (GMM) [11–12]. The modeling methods always require training stage to build up the model. To achieve a better performance, the parameters need to be tuned, such as the number of Gaussian components, the size of training corpus, the selection of acoustic features, the number of HMM states, and so on. The speech sample collection is a bottleneck in the field of CP speech signal processing. The pathological speech data collection is much more difficult than normal speech. In the articles of CP speech signal processing [13–17], the most common sample size is 6–25 participants and 200~500 speech samples. The size of training dataset in HMM/NN/GMM modeling approaches is usually more than 3000 [18]. It is hard to provide such speech samples as training corpus in CP speech for modeling. Moreover, the performance of modeling approaches are subject to the training corpus. If here comes a new kind of utterance which is far from the previous training data, the segmentation accuracy decreases. There are various characteristics of CP speech, including hypernasality, nasal emission, consonant omission, consonant replacement, glottal stop, pharyngeal fricative, pharyngeal stop, posterior nasal fricative, mid-dorsum palatal stop and so on. It is difficult to include all types of CP speech clinical symptoms in training data.

Besides the modeling approaches, a few researches have been done to obtain the Chinese syllable boundaries directly from certain time or frequency domain speech features, such as energy [18–20], power estimated in frequency domain [18], spectrum [18, 21], and Zero-Crossing Rate (ZCR) [22]. The syllable boundaries can often be located roughly, but the exact boundaries are elusive. Zhao [23] and Li [24] have proposed hybrid approaches which utilize multi speech features, and merge multi rounds of boundary selection to improve the segmentation performance. In Zhao's work [23], after the pause detection, the convex hull analysis is applied on short-time energy feature to get the syllable boundaries, and ZCRs are used for boundaries refining. Although a double sliding windowing method is proposed to get more obvious convex hull valleys, the peaks and valleys are still not obvious while using short-time energy feature. It reduces the robustness of Zhao's proposed method. In Li's work [24], the landmarks are set firstly based on estimation of power on frequency domain. Then first round boundaries selection is implemented based on energy and the landmark. The boundaries are refined in the second round using ZCRs. Li has pointed out that some syllable may have two landmarks, for consonant and vowel separately. This mis-segmentation is corrected in the second round segmentation using ZCRs, assuming that vowels have lower ZCRs than initials. However, there are voiced initials in Mandarin, which have similar characteristics of finals. Thus, this system [24] will not be robust for syllable segmentation with voiced initials.

I/F segmentation: Currently, all the I/F segmentation algorithms are implemented on normal speech. Most of current researches achieve the I/F segmentation by investigating the difference of acoustic features between initials and finals, such as autocorrelation [25], Seneff’s auditory spectrum [26], ZCR [27], wavelet transform based features [28], entropy [29] and so on. The I/F boundary can often be roughly located, but accurate segmentation is difficult to implement especially for the syllables with voiced initials. The reason is that the voiced initials have similar characteristics to finals, which makes the I/F boundary blurred. In Mandarin, 4 out of 21 initials are voiced in normal speech. And few existing work has considered the syllables with voiced initials. Li’s work [25] considers this situation, the proposed system detects the voiced speech part firstly, then uses auditory event detection method to classify unvoiced and voiced initials, and locate I/F boundary.

In this work, the automatic I/F segmentation methods in CP speech are proposed. The current researches focus on the speech segmentation in normal speech only. To the best of our knowledge, no work has been done to achieve the automatic I/F segmentation in CP speech. In this work, a hybrid approach is proposed. The speech samples are segmented into syllables firstly, then for each syllable, the proposed I/F segmentation method is implemented.

The rest of this paper is organized as follows. Section 2 describes the background knowledge of Mandarin phonetics, and illustrates the proposed I/F segmentation methods in CP speech. Section 3 presents the experiments and results. The conclusions and discussions are in section 4.

## Structure of a Chinese syllable

A Chinese syllable contains two components: initial and final. The unvoiced/voiced characteristics and time durations of initials/finals are two important clues taking under consideration in I/F segmentation.

Unvoiced/voiced characteristics of initials and finals: the Mandarin initial consonants are unvoiced or voiced, while all the Mandarin finals are voiced [30]. Thus, there are two types of structure model for a syllable: UVI+VF and VI+VF (UVI represents unvoiced initial, VI represents voiced initial, and VF is voiced final). The I/F boundaries for syllables with UVI+VF model are relatively clear, since the acoustic characteristics between unvoiced and voiced speech segments are distinct. However, the I/F segmentation for syllables with VI+VF model is still a problem, since the voiced initials have similar characteristics to voiced finals, which makes the I/F boundaries blur. In this work, a method is proposed to classify the syllables into these two types of structure model firstly. Then different I/F segmentation methods are implemented respectively, in order to improve segmentation accuracy.

Time durations of initials and finals: in the implementation of speech unit segmentation, the time duration of each speech unit is a significant factor. Generally, the time durations of initials are shorter than finals in Mandarin phonetics. The time durations of initials are around 0~200ms in normal speech [25,30]. The Mandarin cleft palate speech follows the general theory of Mandarin phonetics. The time duration of a Mandarin cleft palate syllable is similar to normal speech [31–33]. For many applications of speech signal processing, the length of a speech frame is usually set as 20~30ms [34–37]. To achieve the I/F segmentation in cleft palate speech in this work, considering that some initials are very short, the time duration of a speech frame is chosen shorter than usual frame length to obtain more accurate I/F boundary locations.

## Automatic initial and final segmentation in Mandarin cleft palate speech

**Fig 1:**
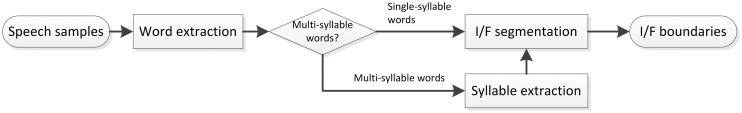
The flowchart of automatic initial and final segmentation system in cleft palate speech.

The proposed system contains two main procedures: syllable extraction and I/F segmentation. (1) Syllable extraction: for the speech utterances, the automatic word detection algorithm is proposed firstly. The detected Mandarin words may contain multiple syllables. For example, one Chinese word might include one, two or three Chinese syllables. The automatic syllable detection algorithm is proposed to extract the syllables in detected words with multi-syllables. (2) I/F segmentation: the extracted syllables are classified into two our self-defined types firstly: syllables with quasi-unvoiced or quasi-voiced initials. Then, for the syllables with quasi-unvoiced initials, the I/F segmentation method is proposed based on wavelet transform, short-time energy and zero crossing rate. For the syllables with quasi-voiced initials, the segmentation method is based on short-time autocorrelation and waveform shape difference between initials and finals. The flowchart of this proposed system is illustrated in Fig 1.

### Automatic syllable segmentation in cleft palate speech

**Fig 2:**
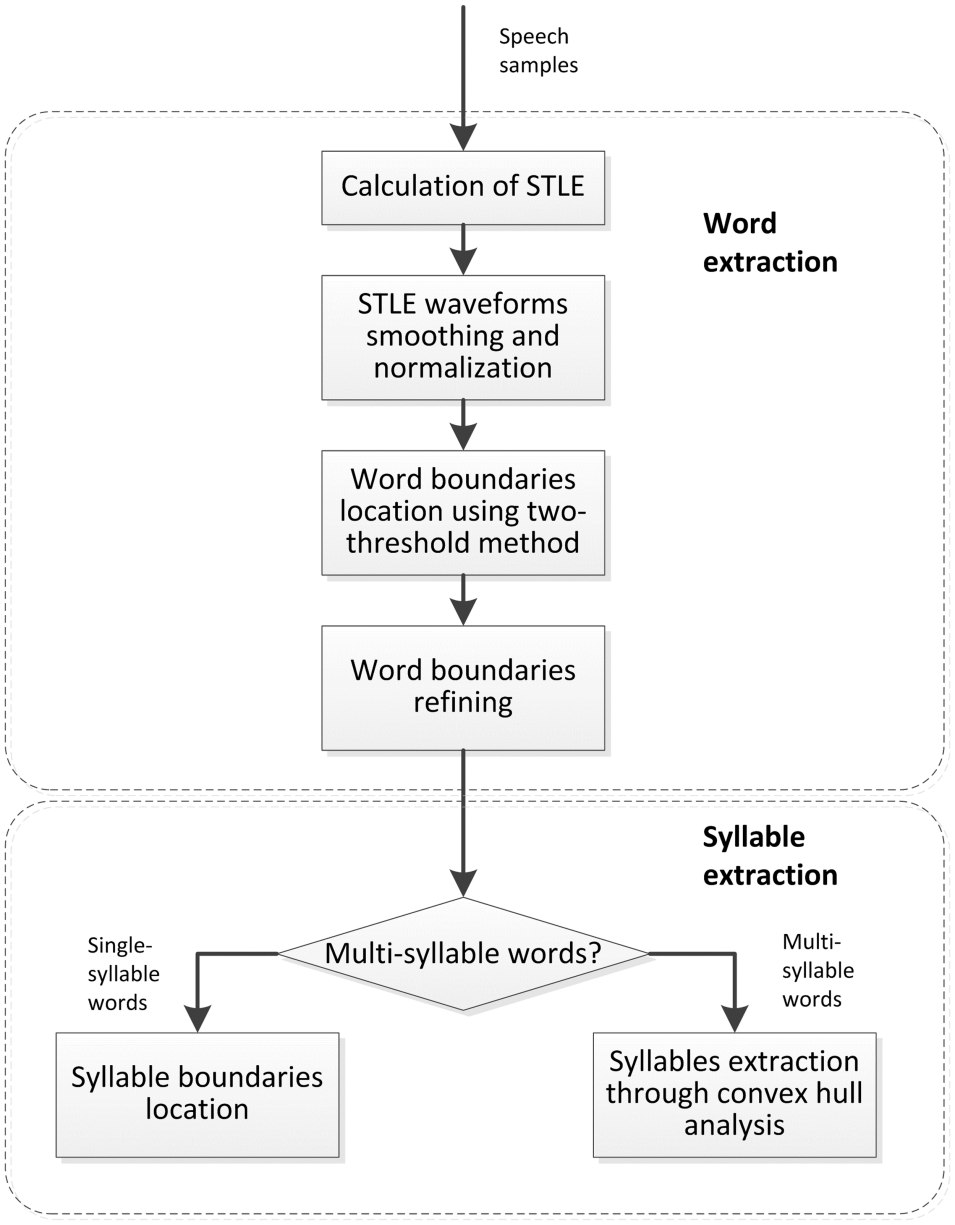
The flowchart of automatic syllable segmentation.

The flowchart of automatic syllable boundaries location in CP speech is listed in Fig 2.

#### Calculation of short-time logarith—Mic energy

Xiao et al. [38] propose a Short-Time Logarith—mic Energy (STLE) parameter, which has a good performance to discriminate speech, noise and silence segments. In this work, STLE is calculated for each framed speech. The frame length is 20ms, with 50% overlap. Suppose that the i^th^ frame speech signal is x_i_(n), STLE is calculated as:

$$L_{i}=\text{lg}(E_{i}+\alpha )-\text{lg}\alpha$$ (1)

$$E_{i}\;=\;\sum_{n=1}^{N}x_{i}^{2}(n)$$ (2)

Where N is the length of speech frame. And *α* is a constant, which is empirically chosen as 5*10^5^, according to the test in [38].

#### STLE waveform smoothing and normalizing

**Fig 3:**
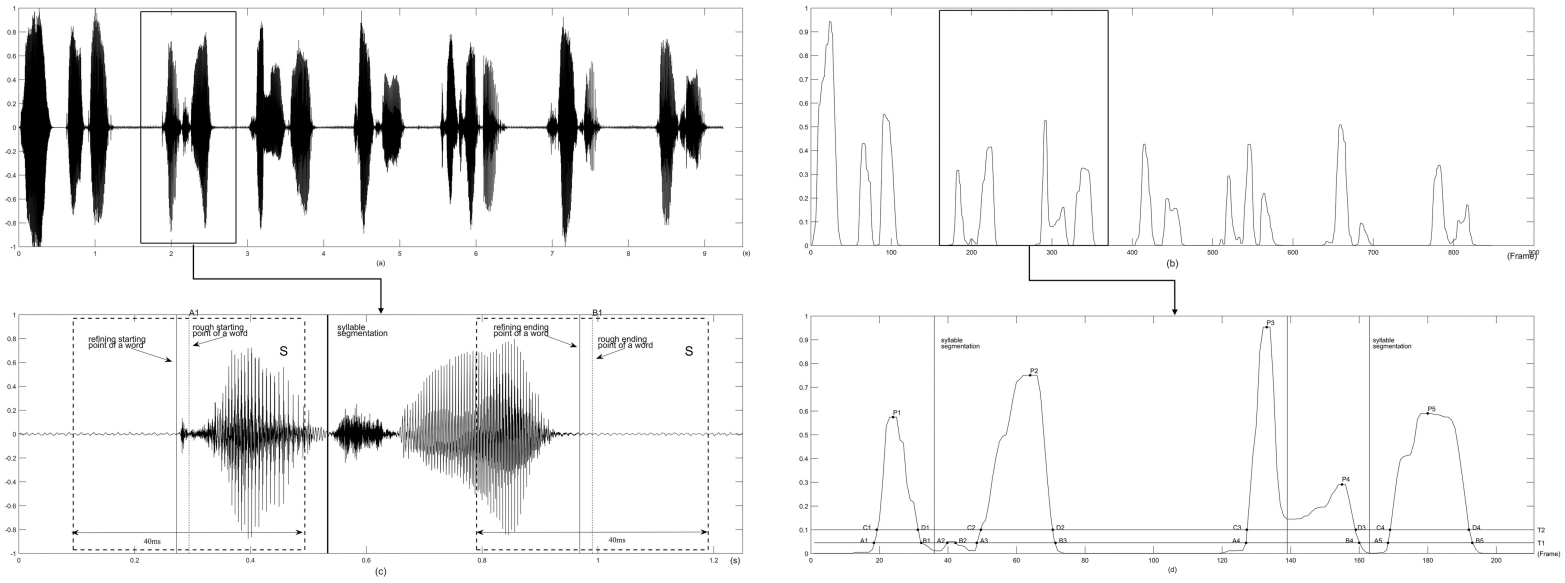
An example of automatic syllable extraction method.

The vector L is smoothed, using the median filter. The filter length is half length of vector L. The smoothed vector is normalized to get vector L’. Fig 3(a) draws the time-domain waveform of a speech utterance spoken by a male cleft palate patient. Fig 3(b) plots the waveform of vector L’.

#### Segmentation of word

A two-threshold method is proposed to detect word boundaries. Fig 3(d) illustrates the word segmentation method. Two threshold T_1_ and T_2_ are set. The intersection points of threshold T_1_ and waveform of vector L’ are the candidate starting (A_i_) and ending (B_i_) frames of the words, i = 1,2,…,N. And the intersection points of threshold T_2_ and waveform of vector L’ are C_i_ and D_i_, i = 1,2,3…M, M≤N. T_1_ and T_2_ are chosen as 0.05 and 0.1 respectively.

These candidates (A_i_ and B_i_) will be deleted, under the following two situations: (1) between the starting frame A_i_ and ending frame B_i_, the maximum amplitude of this signal piece is calculated. If it is less than T_2_, this speech piece is deemed as silence or noise, such as A_2_ and B_2_ as illustrated in Fig 3(d). (2) if the distance between C_i_ and D_i_ is less than 2, which means 2 frames (40ms), this candidate word is deemed as silence or noise as well.

The left A_i_ and B_i_ are rough frame locations of word boundaries, which are illustrated as dashed lines in Fig 3(c).

#### The word boundary refining

The detected word boundaries are further refined. For each starting and ending frame, a piece of speech signal S_i_ is extracted, as illustrated in Fig 3(c). The rough word boundaries are the centers of the extracted speech pieces, which are illustrated as dashed lines in Fig 3(c). The length of the speech piece is 40ms. For this piece of signal, the short-time zero crossing rate is calculated as a vector Z, with 5ms frame length and 50% overlap. Considering the fact that the short-time zero crossing rate of speech part is higher than that of silence part, the jumping point of vector Z should be the boundary of a word. In this work, the vector Z is smoothed using median filter to get the vector Z’. The absolute value of the first order differential of Z’ is calculated, and the position of its maximum value is the boundary frame. The starting point of this frame is the refining location of word boundary, which is illustrated as a solid line in Fig 3(c).

#### Extraction of syllables in multi-syllable words through convex hull analysis

In the above steps, the Chinese words are detected automatically. The detected words might contain one, two or three Chinese characters (syllables). A method is proposed to extract the syllables in multi-syllable words.

For each extracted word, in its waveform of vector L’, the peaks between each pair of A_i_ and B_i_ points are located as P_i_, as marked in Fig 3(d). Then, the minimum point between two adjacent peaks are located as the segmentation frame of syllable. Fig 3(d) illustrated an example of identifying syllable segmentation frame. And the starting point of this frame is the location of syllable boundary, which is illustrated as a solid line in Fig 3(c).

### Automatic initial and final segmentation in cleft palate speech

**Fig 4:**
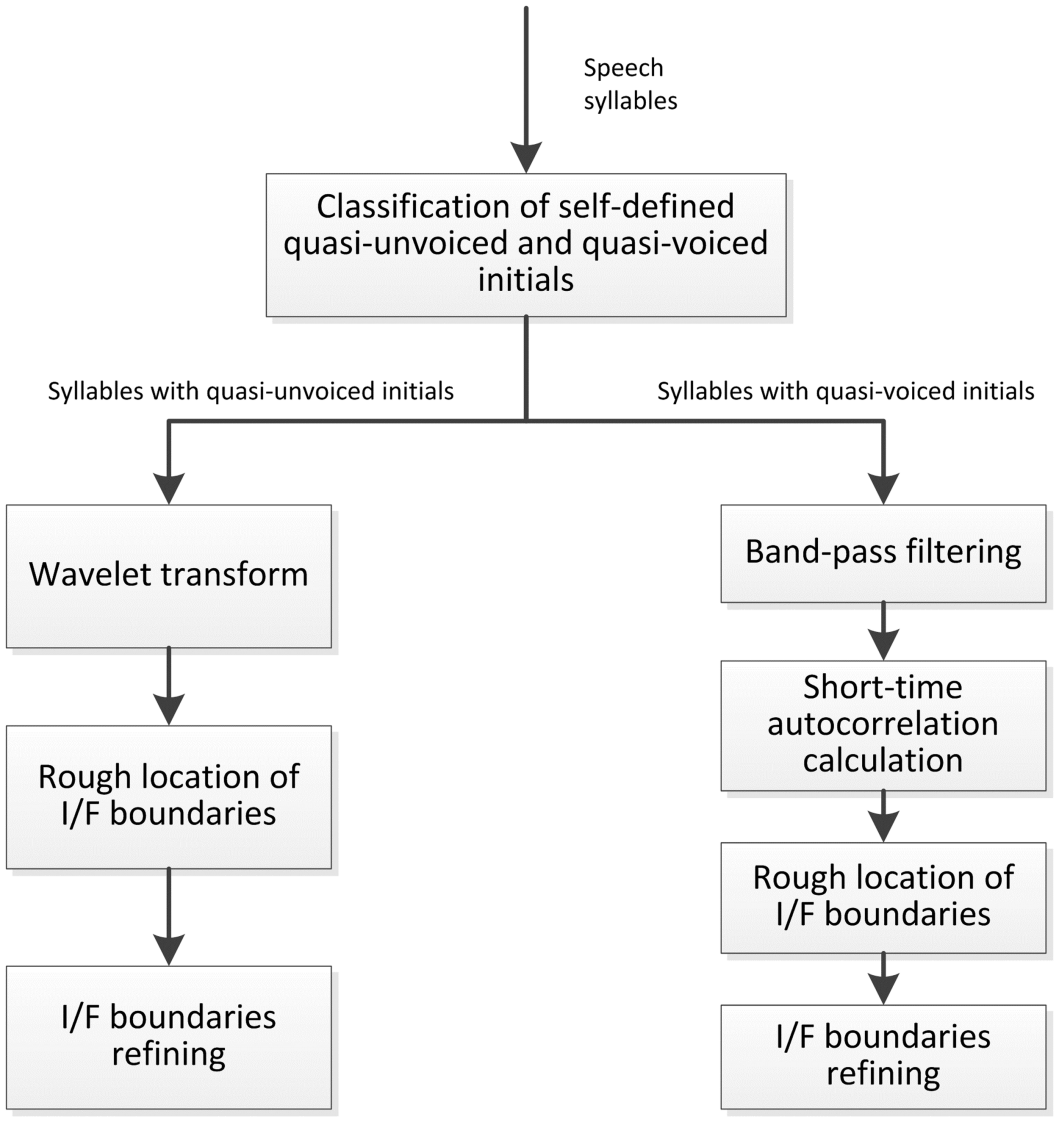
The flowchart of automatic initials/finals segmentation in a Mandarin syllable.

In Mandarin, each syllable is composed of an initial and a final. In this work, a method is proposed to determine whether the initials in the detected syllables are “quasi-unvoiced” or “quasi-voiced” firstly. Then different I/F segmentation methods are applied respectively. The flowchart of I/F segmentation for a Mandarin syllable is listed in Fig 4.

#### Classification of “quasi-unvoiced” and “quasi-voiced” initials

For the normal speech, four initials *m*, *n*, *l*, *r* are voiced, while the left 17 initials are unvoiced. CP patients’ abnormal anatomic structures and wrong pronunciation habits cause alteration of acoustic characteristics in their speech signals. Some voiced initials may represent the characteristics of unvoiced phonemes, while some unvoiced initials may represent the characteristics of voiced phonemes. Thus, the traditional voiced/unvoiced initials classification methods applied to normal speech may not be suitable for cleft palate speech. There are various acoustic characteristics for voiced and unvoiced initials. In this work, quasi-unvoiced and quasi-voiced initials are defined to separate the syllables into two types firstly. Then different I/F segmentation methods are proposed respectively in order to improve the I/F segmentation accuracy.

The voiced phonemes have higher zero-crossing rate than unvoiced phonemes generally. In this work, the syllables are framed with frame length of 20ms, and one third of overlap. The short-time zero-crossing rates for the first 5 frames are calculated as: z_i_, i = 1,2,3,4,5. The time duration of the first 5 frames is 46.7ms, which is a piece of the initial phoneme, considering the time duration of initials in Mandarin [25].

The criterion of syllable classification is as follows:

$$\{\begin{array}{l} \text{max}(z_{i})>50,syllables\;with\;quasi-unvoiced\;initials\\ \text{max}(z_{i})\leq 50,syllables\;with\;quasi-voiced\;initials \end{array}$$ (3)

#### I/F segmentation for syllables with quasi-unvoiced initials

A two-step segmentation method is proposed to get I/F boundaries for syllables with quasi-unvoiced initials: locating the rough I/F boundaries and I/F boundaries refinement.

The first step aims to locate the rough I/F boundaries. For the original speech signal, one-dimensional wavelet decomposition is done, with one layer depth of decomposition, and the mother wavelet is Daubechies2 wavelet. The approximation coefficients and detail coefficients of wavelet decomposition are multiplied to get a new vector C. Then, for the vector C, the short-time absolute amplitudes are summed up, using the following equation:

$$M_{i}=\sum_{k=1}^{N}C_{i}^{}(k)$$ (4)

where i represents the i^th^ frame, and N is the length of frames.

Then a threshold T_3_ is set as 0.005 experimentally. The first intersection point between T_3_ and vector M is the segmentation frame of initial and final. And the starting point of this frame is the segmentation point. The threshold T_3_ determines the rough location of I/F boundaries. These rough boundaries will be refined in the second segmentation step.

**Fig 5:**
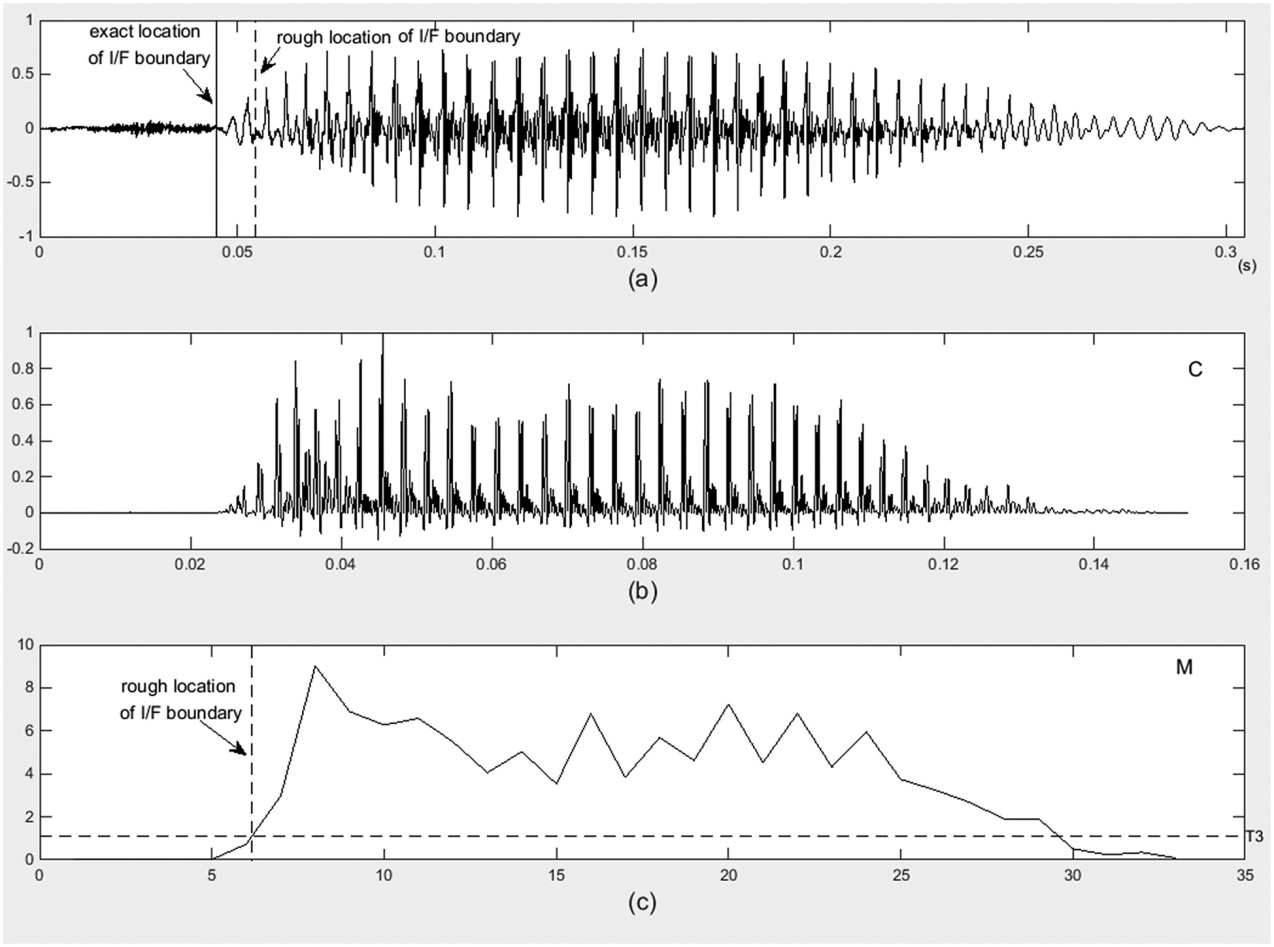
An example of I/F segmentation for syllables with quasi-unvoiced initials.

Fig 5 shows an example of I/F segmentation for syllables with quasi-unvoiced initials. Fig 5(a) draws a time-domain waveform of a speech utterance “ca”, spoken by a female CP patient. Fig 5(b) draws the waveforms of vector C. Fig 5(c) draws the waveform of vector M, the first intersection point of T_3_ and vector M is the rough location of I/F boundary, which is illustrated as a dashed line in Fig 5(a).

The second segmentation step is the I/F boundaries refinement. The boundary refining method is similar to the method listed in section “Automatic syllable segmentation in cleft palate speech”, step (4). For each starting and ending frame, a piece of speech signal is extracted. In this speech piece, the jumping point is located as the refining I/F boundaries. In Fig 5(a), the refined I/F boundary is illustrated as a solid line.

#### I/F segmentation for syllables with quasi-voiced initials

During the pronunciation of voiced initials and all the finals, the glottis vibrates. The acoustic characteristics are similar for both voiced initials and finals, which makes the I/F segmentation for syllables with voiced initials more difficult than unvoiced initials. This work considers the waveform difference between initials and finals, and the time durations of initials. The I/F segmentation steps are as follows:

Band-pass filtering: in this work, the speech signals are band-passed. The cut-off frequencies of band-pass filter are 50Hz and 800Hz. The band-pass filtered signals contain most of semantic information in a speech utterance, and change more slowly than original signals, which makes the I/F segmentation easier through time-domain waveform analysis.

**Fig 6:**
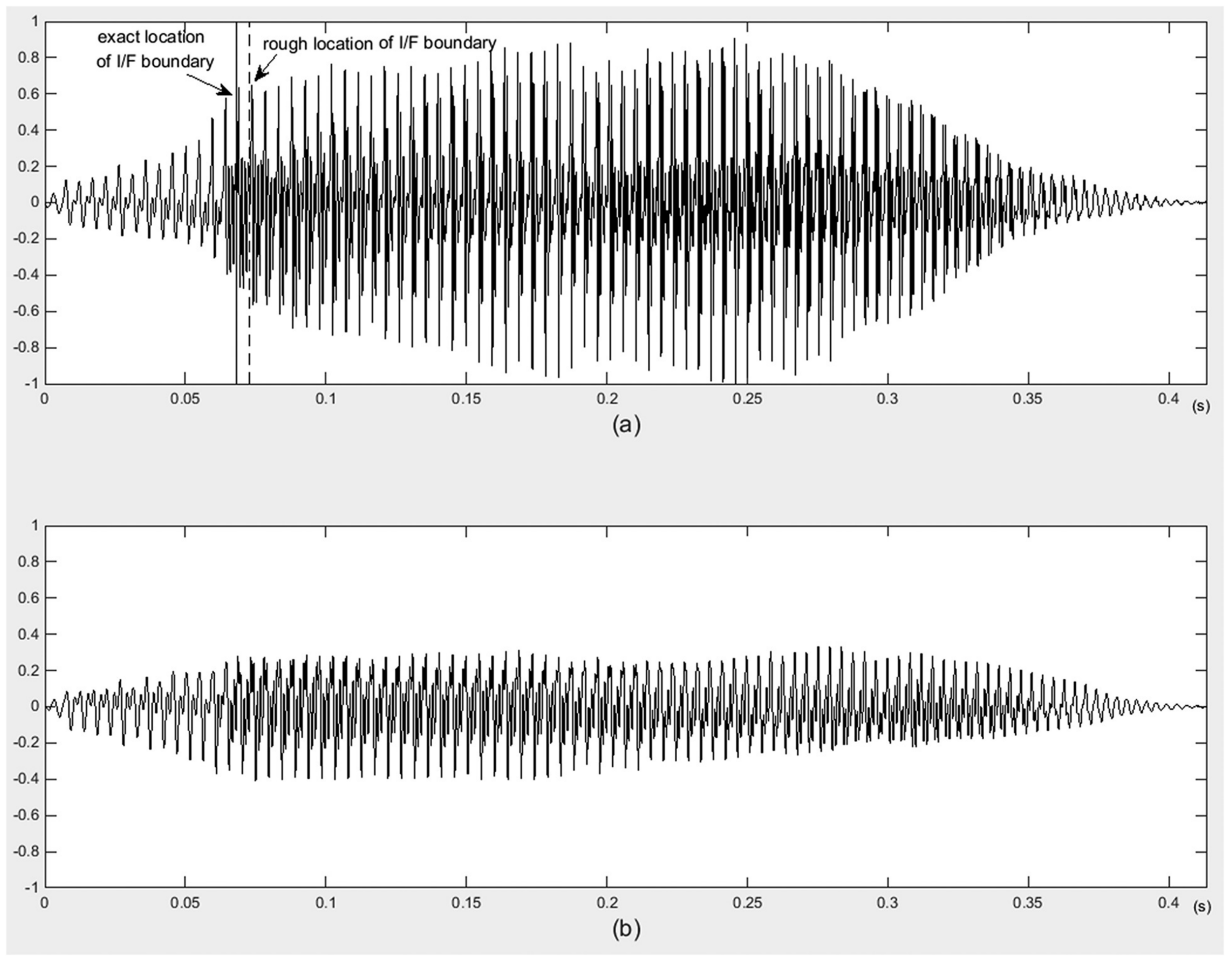
An example of I/F segmentation for syllables with quasi-voiced initials.

Locating rough I/F boundary: the filtered signal is framed, with frame length of 20ms, and two third of overlap. For each speech frame, the number of peaks of the short-time autocorrelation waveform is calculated. The numbers of peaks for each frame form a vector N_t_. Considering the waveform difference between initials and finals, the jumping point of vector N_t_ should be the boundary of initial and final. In this work, vector N_t_ is smoothed using median filter to get the vector N_t_’. The first order differential of N_t_’ is calculated. The frame at the jumping point is the I/F segmentation frame. And the starting point of this frame is the rough location of I/F boundary, which is presented as p. Fig 6 illustrates an example of I/F segmentation for syllables with quasi-unvoiced initials. Fig 6(a) and 6(b) are the time-domain waveform of original and band-pass filtered signals spoken by a male speaker. The dashed lines are the rough locations of I/F boundaries.

I/F boundary refining: an I/F boundary refining method is proposed to obtain more accuracy I/F boundary. A piece of signal centered at point p is extracted, the length of this speech piece is 40ms. For this speech piece, the number of peaks of framed time-domain waveform is calculated. The frame length is 5ms, with 50% overlap. The number of peaks forms a vector N_a_. Vector N_a_ is smoothed using median filter to get the vector N_a_’. The first order differential of N_a_’ is calculated. The frame at the jumping point is the I/F segmentation frame. And the starting point of this frame is the accurate location of I/F boundary. The solid line illustrated in Fig 6(a) is the refined I/F boundary.

## Experiments and results

### Database of cleft palate speech and control samples

#### Cleft palate speech data

The cleft palate speech data are collected by Cleft Palate Speech Treatment Center in the Hospital of Stomatology, Sichuan University. It is the largest cleft lip and palate treatment center in China. The database contains 824 speech segments, including a total of 13080 syllables. The length of each segment is around 10s and the sampling rate is 44100Hz. The speech data are collected from 60 CP patients, including 30 males and 30 females.

The speech database covers typical resonance and articulation disorders in cleft palate speech, including hypernasality, nasal leak, consonant alternative, compensatory articulation and so on. All the speech utterances are assessed independently by three professional speech-language pathologists, and only the speech samples that more than two speech-language pathologists have the consistent idea are selected into this speech database.

The Local Ethics Committee of the Hospital of Stomatology, Sichuan University approves this study and all subjects have given written informed consents prior to the participation.

#### Control samples

The control samples are recorded from 20 normal speakers without cleft palate, including 10 males and 10 females. The control samples contain 228 speech segments, including a total of 3648 syllables. The length of each segment is around 10s and the sampling rate is 44100Hz.

### Experiments and results

#### Syllable segmentation results

The automatic syllable segmentation results are compared with the manually segmentation results. The tolerance is 20ms. If both the shift of starting and ending boundaries of a syllable are less than 20ms, this syllable is counted as correct labels.

The syllable extraction accuracy is defined as:

$$P_{S}=\frac{N_{S}}{N_{SA}}$$ (5)

Where P_S_ is the detection accuracy of syllable. N_S_ is the number of syllables which are correctly extracted. N_SA_ is the total number of syllables. In this experiment, P_S_ is 90.62% for cleft palate speech data, and 93.93% for control samples.

#### I/F segmentation results

The automatically extracted syllables are further processed for I/F segmentation. The automatic I/F segmentation results are compared with the manually segmented results. The time errors between the automatic segmentation results and the gold standard are listed in Table 1. Many current researches [25,26,28,39–43] calculate the correct I/F segmentation percentage, which is defined as:

$$P_{I/F}=\frac{N_{I/F}}{N_{S}}$$ (6)

where N_I/F_ is the number of syllables whose time shifts between automatic and manual segmentation results are less than t millisecond. N_S_ is the total number of syllables. The time tolerance is usually set as 20ms. In this work, P_10_, P_20_ and P_30_ are calculated and listed in Table 1.

**Table 1:** The I/F segmentation accuracy for cleft palate speech data and control samples.

	syllables with quasi-unvoiced initials		syllables with quasi-voiced initials		All syllables	
	Cleft palate speech	Control samples	Cleft palate speech	Control samples	Cleft palate speech	Control samples
Mean time errors (ms)	4.4	5.3	25.7	32.1	9.6	10.6
Deviation of time errors (ms)	12.3	14.7	56.4	64.2	31.1	24.5
P_10_ (%)	91.24	89.77	61.86	58.21	84.14	82.86
P_20_ (%)	94.52	93.44	70.3	74.63	88.68	89.24
P_30_ (%)	96.22	95.27	77.47	76.21	91.69	91.24

From Table 1, it is seen that the proposed method achieves higher segmentation accuracy for syllables with quasi-unvoiced initials than that of quasi-voiced initials, for both cleft palate speech and control samples. Overall, the I/F segmentation accuracies P_30_ for all syllables are 91.69% for CP speech and 91.24% for normal speech. The I/F segmentation accuracies P_10_ and P_20_ are lower than P_30_, but are still over 80% for both cleft palate speech and control samples with all the syllables.

#### Performance comparisons with state-of-the-art methods

Automatic syllable segmentation results: currently, the syllable segmentation methods are mainly based on the following speech features: short-time ZCR, short-time energy, short-time amplitude [19, 22], wavelet transformation combined with entropy [20], spectrum entropy in specific frequency bands [21], double sliding window energy combined with short-time ZCR [23], power in frequency domain combined with short-time ZCR [24].

The syllable extraction accuracies P_S_ are calculated for both cleft palate speech data and control samples, using state-of-the-art methods and our proposed method. The results are listed in Table 2.

**Table 2:** The syllable extraction accuracies using state-of-the-art methods and our proposed method (%).

	short-time ZCR + energy + amplitude [19, 22]	wavelet transformation + entropy [20]	Spectrum entropy + filtering [21]	double sliding window energy + short-time ZCR [23]	Power + filtering + short-time ZCR [24]	Our proposed method
Cleft palate speech	50.16	56.34	50.30	75.6	78.3	90.62
Control samples	60.13	70.97	74.19	90.5	94.2	93.93

Table 2 shows that both state-of-the-art methods and our proposed method achieve higher syllable extraction accuracies for normal speech than that for cleft palate speech. Moreover, our proposed method achieves higher syllable segmentation accuracy in cleft palate speech than using state-of-the-art methods.

Automatic I/F segmentation results: the current I/F segmentation studies are mainly based on the following algorithms: auditory model [25, 26, 35], short-time energy, short-time amplitude, short-time ZCR [27], discrete wavelet transform [28], entropy [29], auditory event detection [39], short-time energy in specific frequency band [40]. In the experiment, the I/F segmentation accuracies are tested on cleft palate speech data and control samples, using state-of-the-art methods and our proposed method. The experimental results P_30_ are calculated and listed in Table 3.

**Table 3:** The I/F segmentation accuracies P_30_ using state-of-the-art methods and our proposed method (%).

	auditory model [25, 26, 35]		short-time energy + amplitude + ZCR [27]		discrete wavelet transform [28]		entropy [29]		auditory event detection [39]		short-time energy + filtering [40]		Our proposed method	
	UV^a^	V^b^	UV^a^	V^b^	UV^a^	V^b^	UV^a^	V^b^	UV^a^	V^b^	UV^a^	V^b^	UV^a^	V^b^
Cleft palate speech	78.5	62.8	71.4	75.5	92.8	61.0	60.0	50.3	68.5	52.8	78.4	64.2	96.2	77.5
Control samples	88.2	69.1	89.1	68.7	94.1	63.7	63.7	55.0	78.2	59.1	88.2	68.7	95.3	76.2

^a^UV: syllables with unvoiced initials.

^b^V: syllables with voiced initials.

As seen from Table 3, state-of-the-art methods obtain lower I/F segmentation accuracies for cleft palate speech than that for control samples. Our proposed method achieves higher I/F segmentation accuracy in cleft palate speech than using state-of-the-art methods.

Table 3 also shows that most of state-of-the-art methods achieve a good performance on I/F segmentation for the syllables with unvoiced initials. However, the segmentation accuracies decrease for the syllables with voiced initials. The proposed method is more robust for syllables with both unvoiced and voiced initials than state-of-the-art methods.

## Conclusions and discussions

The occurrence of cleft palate and lip is 0.182% in China. China has the largest number of CP patients. The implement of an automatic CP speech assessment system could provide assisted aids to speech-language pathologists and patients. The speech unit segmentation is an important pre-processing step in CP speech analysis. Although many researches have been done to implement I/F segmentation in normal Mandarin speech, rare research has been done to investigate I/F segmentation methods in CP speech. The difficulty of CP speech collection and annotation is a major reason. The collection and annotation of CP speech database usually takes years of time, and requires high professionalism of speech-language pathologists. In this work, the I/F segmentation methods are investigated based on an intensive CP speech database. The I/F segmentation is implemented in two steps: syllables segmentation and I/F segmentation.

For the syllable segmentation, the speech utterances are segmented into words firstly, based on the Short-Time Logarith—mic Energy (STLE) feature, which provides more obvious convex hull peaks and valleys than short-time energy feature applied in article [38]. A two thresholds algorithm is proposed on STLE contour to get the rough word boundary, combining with the Mandarin phonetics information. The word boundaries are refined using ZCRs. Then, if the detected word contains multi-syllables, each syllable is segmented through convex hull valley analysis. Compared with the power feature applied in article [24], the STLE feature is not sensitive to voiced or unvoiced speech. In this experiment, the proposed algorithm is simpler than existing modeling approaches. It could be efficiently applied in real-time application. Moreover, the proposed method requires no training stage, which needs thousands of training samples in modeling approaches. In current research articles, the syllable segmentation accuracy for normal speech is usually around 75%-95%. The speech production process and acoustic characteristics of pathologic speech are more complex than normal speech. In this work, the accuracy of syllable segmentation in CP speech is 90.62%.

For the extracted syllables, the I/F segmentation methods are applied. There are 21 initials in Mandarin. Except 4 initials *m*, *n*, *l*, *r* are voiced, the left 17 initials are unvoiced. The finals are composed of vowels or compound vowels [30]. All the finals are voiced. Thus, the voiced and unvoiced characteristic of speech segments is a crucial factor in I/F segmentation. The majority of current articles [26–29] study I/F segmentation for syllables with unvoiced initials only, considering that there are only 4 out of 21 voiced initials in normal speech. The structure of a Mandarin syllable is simplified into the UVI+VF model. The acoustic characteristics between unvoiced and voiced speech segments are distinct. Therefore, the I/F segmentation performances of those articles are good for syllables with unvoiced initials, but the segmentation accuracies decrease for syllables with voiced initials. Few article [25] has studied the I/F segmentation for syllables with both unvoiced and voiced initials in normal speech. For the cleft palate patients, their abnormal anatomic structures and wrong pronunciation habit might change some speech characteristics, including the unvoiced and voiced characteristics of speech segments. The simplification of a Mandarin syllable structure is no longer suitable for the cleft palate speech processing. In this work, the unvoiced and voiced characteristics of initials are fully considered in the process of I/F segmentation. Quasi-unvoiced and quasi-voiced initials are defined in this work, whose characteristics are similar to the unvoiced and voiced initials in normal speech respectively. To improve the efficiency and segmentation accuracy, the syllables are classified into two types firstly by calculating ZCRs: syllables with quasi-unvoiced or quasi-voiced initials. Then respective I/F segmentation methods are proposed to those two types of syllables. Compared with Li’s work [25], the method of voiced/unvoiced initials classification is less complex and more efficient. Moreover, in order to improve the robustness of segmentation accuracy, the two-step segmentation method is proposed. The rough location of I/F boundary obtained in the first-step is refined in the second-step procedure to get a more accurate segmentation location.

For the syllables with quasi-unvoiced initials, the mean time error is around 4ms. While for the syllables with quasi-voiced initials, the segmentation performance decreases, and the mean time error is around 25ms. The quasi-voiced initials present similar acoustic characteristics to the finals, which leads to a lower I/F segmentation accuracy.

The current I/F segmentation methods in Mandarin are implemented on normal speech. Most of these researches are based on limited size of speech database. The testing speech samples are usually around 200~400 spoken by less than 10 speakers [40,41,43]. Only few work have tested speech samples more than 1000 syllables [25,42]. The speech data tested in this work is intensive, it contains around 3000 Chinese syllables.

The current I/F segmentation methods usually take P_18~35_ as the segmentation accuracies, which are around 84~93%. If taking P_30_ in this work, the segmentation accuracy is 91.69% for all the syllables, and the accuracy reaches up to 96.22% for the syllables with quasi-unvoiced initials, while it is 77.47% for quasi-voiced initials.

The proposed method is tested on both CP speech and control samples (normal speech spoken by people without cleft palate). The results listed in Table 1 show that the proposed method has a good performance on I/F segmentation for both cleft palate speech and control samples. Although there are differences of acoustic and prosodic characteristics between cleft palate and normal speech, the presented method proposes following methods to improve algorithm’s robustness. This work proposes separate processing methods for syllables with quasi-unvoiced/voiced initials, in order to improve I/F segmentation accuracy. Moreover, this work proposes a two-round segmentation method to obtain more accurate I/F boundaries.

In this work, state-of-the-art methods are tested for comparison purpose. Tables 2 and 3 list Mandarin syllable and I/F segmentation accuracies using state-of-the-art methods and our proposed method. The experimental results show that state-of-the-art methods obtain better performances for normal speech than that for cleft palate speech. The existence of resonance disorders and articulation disorders changes some characteristics of cleft palate speech. The current segmentation algorithms implemented on normal speech are not suitable for cleft palate speech. The proposed method considers characteristics of cleft palate speech, and achieves higher syllable and I/F segmentation accuracies for cleft palate speech than using state-of-the-art methods.

Despite that the languages across the world are disparate in phonetic contents and linguistic rules, their phoneme composition rules are similar. A syllable of any language can be viewed as a certain configuration of vowels and consonants [44]. The clinical symptoms of cleft palate speech are similar in almost all the languages. Thus, this work could be a reference to cleft palate speech phoneme segmentation in different languages.

## Supporting information

S1 File Min speech data. (RAR)
